# 

**Published:** 1870-06

**Authors:** 


### *Graduates of Medical Colleges for 1870,*

Corrected from the *New York Medical Journal* of May, 1870:

| | |
|---|---|
| Jefferson Medical College, Philadelphia | 163 |
| Bellevue Hospital Medical College, New York | 140 |
| Rush Medical College, Chicago | 131 |
| University of Pennsylvania, Philadelphia | 113 |
| University of Louisville, Ky | 92 |
| College of Physicians and Surgeons, N. Y | 70 |
| University Medical College, New York | 62 |
| Nashville Medical College, Tenn | 58 |
| Washington University, Baltimore | 48 |
| Buffalo Medical College, New York | 41 |
| Massachusetts Medical College, Boston | 39 |
| Miami Medical College, Cincinnati | 37 |
| Albany Medical College, New York | 28 |
| Starling Medical College, Columbus, O | 24 |
| Chicago Medical College, Illinois | 20 |
| Toland Medical College, San Francisco | 9 |

